# The Evolution of Bacterial Genome Architecture

**DOI:** 10.3389/fgene.2017.00072

**Published:** 2017-05-30

**Authors:** Louis-Marie Bobay, Howard Ochman

**Affiliations:** Department of Integrative Biology, University of Texas, AustinTX, United States

**Keywords:** genetic drift, genome, bacterial, genome evolution, horizontal gene transfer, population dynamics

## Abstract

The genome architecture of bacteria and eukaryotes evolves in opposite directions when subject to genetic drift, a difference that can be ascribed to the fact that bacteria exhibit a mutational bias that deletes superfluous sequences, whereas eukaryotes are biased toward large insertions. Expansion of eukaryotic genomes occurs through the addition of non-functional sequences, such as repetitive sequences and transposable elements, whereas variation in bacterial genome size is largely due to the acquisition and loss of functional accessory genes. These properties create the situation in which eukaryotes with very similar numbers of genes can have vastly different genome sizes, while in bacteria, gene number scales linearly with genome size. Some bacterial genomes, however, particularly those of species that undergo bottlenecks due to recent association with hosts, accumulate pseudogenes and mobile elements, conferring them a low gene content relative to their genome size. These non-functional sequences are gradually eroded and eliminated after long-term association with hosts, with the result that obligate symbionts have the smallest genomes of any cellular organism. The architecture of bacterial genomes is shaped by complex and diverse processes, but for most bacterial species, genome size is governed by a non-adaptive process, i.e., genetic drift coupled with a mutational bias toward deletions. Thus, bacteria with small effective population sizes typically have the smallest genomes. Some marine bacteria counter this near-universal trend: despite having immense population sizes, selection, not drift, acts to reduce genome size in response to metabolic constraints in their nutrient-limited environment.

## Introduction

The overall structure and organization of bacterial genomes were well resolved before the golden era of genome sequencing. It was known that bacterial genomes varied in size by at least an order of magnitude and even that there could be considerable variation in genome size within a bacterial species (Herdman, 1985); that bacterial genomes typically comprised one circular chromosome but often harbored extrachromosomal elements in the form of plasmids or phages (Lederberg, 1998); that base composition was relatively uniform along the chromosome but highly variable across species, ranging from 13 to 75% G + C (Thomas et al., 2008; McCutcheon and Moran, 2010); that bacterial genomes consisted mostly of functional protein-coding regions, with little non-coding or intervening sequences (Mira et al., 2001); that genetic maps (and hence, gene order and gene content) remained fairly stable among related species (Rocha, 2008); that genome architecture could be altered by insertions, duplications, inversions, and translocations, fostered, in part, by mobile elements (Eisen et al., 2000; Tillier and Collins, 2000; Rocha, 2008); and that the bacterial chromosome is configured into domains that relate to its replication and packaging (Boccard et al., 2005).

Many of these features of bacterial genomes contrast those of eukaryotic genomes, which are often partitioned into multiple linear chromosomes and are generally much larger due both to increases in gene number and to the proliferation of non-coding and repetitive DNA. Although the properties of genomes and the variation in genome architecture across the tree of life were recognized by cytogeneticists and molecular biologists alike, it was not until large numbers of bacterial genome sequences became available that the processes underlying their evolution could be fully appreciated.

## Microevolutionary Processes Drive Genome Architecture

Like other biological features, the mechanisms forging the content and organization of bacterial genomes rely on selection and drift, whose relative contributions are dictated by the effective population size (N_e_) and the selection coefficient (*s*) associated with a trait (Wright, 1931; Kimura, 1968). In a haploid organism, evolution is driven by stochastic processes (i.e., drift) when |*2×Ne×s*|<<1, whereas selection dominates when |*2×Ne×s*| >>1 (Kimura, 1968). This central concept of population genetics was further expanded under the nearly neutral theory of evolution of Ohta (1973), who put forward the notion that, although selection does not change, the response to selection depends on the effective size of populations. Indeed, the selection coefficient *s* is a variable parameter that only depends on the impact of a given gene variant on the fitness of the individual relative to others in the population. In contrast, the long-term effective population size is a parameter that influences the impact of selection relative to drift, since smaller populations are more strongly affected by the random sampling of genotypes at each generation. Note that at the extremes, such that a trait is either essential (i.e., its disruption is lethal) or completely neutral (i.e., its disruption is inconsequential), effective population size does not affect its fixation; however, the fate of all other variants, and of the vast majority of sequences in a bacterial genome, depends on the interplay of selection and drift.

Considering these factors, it has been proposed that the architecture of genomes varies as a function of the effective population size (N_e_) and the mutation rate (μ), under the so-called “mutational hazard hypothesis” (Lynch and Conery, 2003; Lynch et al., 2011). Those species with small effective population sizes, such as many animals and plants, will experience strong effects of drift-guided evolution and accumulate large amounts of moderately deleterious DNA, including mobile elements, pseudogenes, and introns (Lynch et al., 2011). In humans, whose effective population size is estimated to be lower than 10,000 (Takahata, 1993; Tenesa et al., 2007), sequences encoding functional proteins represent only <5% of genomic DNA, due to the genome-wide expansion of numerous genetic elements, such as introns, LINEs, and SINEs. Amassing these sequences is thought to represent a substantial mutational burden, since intron splice sites can represent potential targets for mutations and each new mobile element can potentially insert into and disrupt a functional region (Lynch, 2002; Lynch et al., 2011). In contrast, species with large effective population sizes evolve predominantly through selection, thereby preventing the accumulation of hazardous elements.

Relative to multicellular organisms, bacteria exhibit small, gene-rich genomes, typically under 10 Mb in length (Kuo et al., 2009). At first glance, these features seem to fit with the mutational hazard hypothesis, such that the large population sizes of bacteria increase the efficacy of selection, which fosters the removal of deleterious sequences and results in compact genomes consisting mostly of the functional genes (Lynch, 2006). However, the trend in bacteria actually runs *opposite* to the predictions of the mutational hazard hypothesis (Daubin and Moran, 2004; Kuo et al., 2009): bacterial species with the lowest effective population sizes, such as endosymbiotic bacteria whose effective population sizes approximate those of their animal hosts, typically have the smallest and most compact genomes, whereas those with the largest populations exhibit the expansive genomes (Kuo et al., 2009). This circumstance raises questions about why the genomic trends in bacteria differ from those of eukaryotes; and in this review, we resolve the population-level parameters as well as the mutational mechanisms that shape the structure, content, and evolution of bacterial genomes.

## Defining Bacterial Species and Populations

Due to their unicellularity and uniformity in genome structure, bacteria are typically viewed as simple organisms. However, many of the most basic features of their populations remain obscure, often making it difficult to evaluate and quantify microevolutionary processes. The first issue surrounds the definition of a bacterial species (Shapiro et al., 2016). Sexual organisms are usually classified into species that represent units that are genetically and phenotypically cohesive, and the most widely applied species definition—the Biological Species Concept—allows for a simple and uniform classification of species across all sexual organisms (Mayr, 1942). The delineation of bacterial species is much more problematic, since no biologically relevant species concept is appropriate for asexual organisms that sporadically exchange or acquire genes by recombination or lateral gene transfer (Shapiro and Polz, 2014, 2015). Different conceptual frameworks, such as the ecotype definition, have been proposed (Cohan, 2001) but are difficult in practice to apply. In contrast, sequence-similarity thresholds are easy to apply but need not be biologically relevant (Konstantinidis and Tiedje, 2005; Hugenholtz et al., 2016; Bobay and Ochman, 2017). Estimation of several population genetic parameters relies on assessments of the allelic variation in conspecifics, so the arbitrary assignment of bacterial strains to species can (and has) lead to many contradictory conclusions about bacterial evolution.

Apart from delineation of species, the estimation of effective population sizes (N_e_) is difficult in bacteria, both because they are difficult to observe and because they violate some of the assumptions of the Wright–Fisher model (Hartl and Clark, 2007). Aside from those few host-associated bacteria whose transmission dynamics are known, estimates of N_e_ for most bacterial species vary over several orders of magnitude depending on how and which populations are being assessed. Genomic-based strategies for estimating N_e_ are usually based on the extent of genomic diversity at neutral sites. N_e_ for haploid organisms is given by *𝜃 = 2×N_e_×*μ (Watterson, 1975), where *𝜃* is the number of segregating sites and μ is the mutation rate. The existence of truly neutral sites in bacteria has been called into question, since codon usage and nucleotide composition appear to be under weak selection in many species (Rocha and Feil, 2010). If this is the case, estimates based on such metrics should be considered prudently, especially in those species with large population sizes, since the effectiveness of selection at such sites would be enhanced as N_e_ becomes larger.

Estimating *𝜃* may be confounded by the fact that bacteria reproduce clonally, and the linkage of alleles makes them highly susceptible to Hill–Robertson effects (i.e., background selection, hitchhiking, and Muller’s ratchet; Hill and Robertson, 1966; Felsenstein, 1974; Smith and Haigh, 1974; Charlesworth et al., 1993), such that selection on a beneficial or detrimental allele in a given genotype will lead to the loss of allelic diversity. Because deleterious mutations are expected to be frequent, it has been predicted that background selection leads to the loss of substantial genetic diversity in bacterial populations (Betancourt et al., 2009; Price and Arkin, 2015). It is important to note, however, that very few bacteria are truly clonal and that most engage in some homologous recombination (Vos and Didelot, 2009), which liberates alleles from genomic linkage and counteracts Hill–Robertson effects (Betancourt et al., 2009). Unlike recombination, whose rate is unpredictable for a given bacterial species, it is thought that μ is relatively constant across species. Mutation rates are fairly similar in most of the 10 or so bacterial species that have been assayed in the laboratory; however, they are still unknown for the vast majority of bacterial species and can vary up to 100-fold (Sung et al., 2016). Together, these factors make estimations of N_e_ based on the neutral expectations an imperfect metric.

A more convenient though indirect measure of N_e_ is based on assessment of K_a_/K_s_ or *d_N_/d_S_*Kindly advise whether “K_a_/K_s_ or *dN/ds*” should be changed to “*K_*a*_/K_*s*_* or *d_*N*_/d_*s*_*” here and throughout. ratios, which represent the effectiveness of selection and scale negatively with N_e_, since smaller populations promote the fixation of slightly deleterious mutations thereby increasing *K_a_* (or *d_N_*) (Daubin and Moran, 2004; Kryazhimskiy and Plotkin, 2008). Although **d_N_/d_S_** ratios are not constant over time when computed on genomes of the same species (Rocha et al., 2006; Kryazhimskiy and Plotkin, 2008) and can vary when genes are under different selective constraints (Batut et al., 2014), it provides a more robust metric for comparing N_e_ across species when adjusted for divergence times (e.g., by applying *dS* thresholds) and limited to comparisons of identical sets of genes in different species.

When analyzed across a diverse array of taxa, K_a_/K_s_ ratios proved to be a fairly reliable proxy for N_e_, since the values seemed to fit with what was known about the natural history of the specific bacterial groups. For example, endosymbiotic, parasitic, and other obligatory host-associated bacteria displayed high K_a_/K_s_ ratios and are known to have effective population size that are small, approximating those of their animal hosts. In contrast, broadly distributed, environmental bacteria, presumed to have very large effective population sizes, displayed the lowest K_a_/K_s_ ratios. It was also determined that K_a_/K_s_ ratios scaled with genome size, such that bacteria with higher values (i.e., smaller N_e_) have more highly reduced genomes, and this association holds across phylogenetically divergent bacteria (Kuo et al., 2009).

## How Large are the Effective Population Sizes of Bacteria?

Although the estimation of N_e_ is challenging, studies based on nucleotide diversity at neutral sites suggest that most bacterial species have an effective population size in the range of 10^6^–10^9^ (Sung et al., 2012). However, estimates based on **d_N_/d_S_** ratios—but including some additional species—yielded average estimates ranging from 10^6^ to 10^12^ (Sela et al., 2016). It is surprising that the most abundant species on the planet, the marine bacterium *Prochlorococcus*, was estimated to have an N_e_ of only 1.5×10^9^, since based on its census population, N_e_ could reach 10^13^ in this “species” (Kashtan et al., 2014). The N_e_ estimated from allelic diversity is likely an underestimation, as might occur if synonymous positions are not strictly neutral. But because the population dynamics of *Prochlorococcus* is largely unknown, it is possible that N_e_ is indeed much lower than the census population size due to frequent and drastic demographic variations, such as genotype sweeps and bottlenecks.

On the other end of the spectrum, endosymbionts experienced strong reductions in population sizes. Being confined within the cells of their hosts, and in the most extreme cases, transmitted by exclusively maternal lines, endosymbionts experience severe bottlenecks during propagation (Moran, 1996; Moran et al., 2009). In the aphid endosymbiont *Buchnera aphidicola*, N_e_ was estimated to be ∼10^6^ (Funk et al., 2001; Moran et al., 2009), but its mutation rate has not been directly estimated in the lab. The only small-genomed bacterium whose mutation rate has been accurately measured is the intracellular bacterium *Mesoplasma florum*, and its N_e_ was also estimated to be 10^6^ (Sung et al., 2012), again among the lowest determined for bacteria.

## The Mutational Hazard Hypothesis and Bacteria

Because genome size in bacteria scales positively with N_e_, bacteria defy the predictions of the mutational hazard hypothesis. Bacteria tend to have larger genomes when selection is more effective (Kuo et al., 2009; Sela et al., 2016), whereas eukaryotes have more streamlined genomes when selection is more effective (Lynch and Conery, 2003; Lynch et al., 2011). This raises a paradox as to how and why the same force leads to opposite effects in bacteria and eukaryotes.

The answer resides in differences in the mutational processes: in bacteria, there is a strong mutational bias toward deleting superfluous sequences (Andersson and Andersson, 2001; Mira et al., 2001). It has long been known that gene number increases linearly with genome size in bacteria and that pseudogenes are rare or absent from bacterial genomes. This contrasts that situation in eukaryotic lineages in which there is little correlation between genome size and gene number—the “C-value paradox”—and there are pseudogenized copies of most genes (Lynch, 2007). In bacteria, deletional bias is apparent at all levels of genome organization: individual strains in culture incur large deletions encompassing up to 5% of their genome (Nilsson et al., 2005), comparisons of pseudogenes to their functional counterparts show that inactivated regions perpetually erode by small deletions (Mira et al., 2001; Kuo et al., 2009), and broad phylogenetic comparisons indicate that lineages of host-associated bacteria with small genomes derive from ancestors with large genomes over evolutionary timescales (Ochman, 2005).

The reason that bacterial species undergoing less effective selection (i.e., lower N_e_) have smaller genomes is that they have accrued and tolerated more deleterious mutations due to drift. This is particularly evident in the genomes of pathogens and symbionts since their host-associated lifestyle both increases the fixation of slightly deleterious mutations and renders many previously useful genes redundant in the nutrient-rich host environment, thereby generating large numbers of non-essential regions that are subsequently removed by the pervasive mutational bias toward deletions. Note that the primary force countering gene erosion and elimination is natural selection, with the result that bacterial genomes, both large and small, maintain a high density of functional sequences (Ochman and Moran, 2001).

Genetic drift, coupled with deletional bias, are major determinants of bacterial genome size, such that species with the smallest N_e_ have the smallest genomes. But some—the marine bacteria—do not follow this trend and represent a curious exception. Marine bacteria have very large census population sizes but possess highly reduced genomes, on the order ∼1.5 Mb in length (Giovannoni et al., 2014; Kashtan et al., 2014). Moreover, these genomes harbor the smallest amount of intergenic DNA, with a median spacer length of only 3 bp between coding regions (Giovannoni et al., 2005). It has been hypothesized that genome reduction in marine species results from the efficacy of selection that can only occur in extremely large populations: these organisms live in nutrient-limited environments such that elimination of each non-essential nucleotide imparts an advantage by reducing the metabolic costs associated with DNA replication and processing (Giovannoni et al., 2014). In most populations, fitness differences this small would not be discriminated by selection; however, marine species provide a special case where selection, not genetic drift, governs genome size reduction.

## Effects of Population Size on Genome Content and Complexity

The linear relationship between genome size and gene number in bacteria implies that the proportion of non-coding and intergenic DNA is the same in all genomes. The effects of population size are also evident on bacterial genome complexity, i.e., the number and fraction of functional genes in a genome. Whereas intergenic regions typically constitute 10 ± 5% of a bacterial genome, species subject to drift sometimes can have much greater amounts of DNA that do not specify functional proteins. In particular, the genomes of bacteria that have sustained episodes of strong reductions in population size, such as pathogens and symbionts have recently become associated with hosts, contain large numbers of pseudogenes and/or mobile elements.

Most bacterial genomes maintain very low numbers of insertion sequence (IS) elements (<10; Touchon and Rocha, 2007) whereas several recent pathogens (e.g., *Shigella* spp. and *Rickettsia* spp.; Fuxelius et al., 2007; Touchon et al., 2009) and symbionts (e.g., *Sodalis glossinidius* and *Serratia symbiotica*; Toh et al., 2006; McCutcheon and Moran, 2012; Manzano-Marin and Latorre, 2014) possess hundreds of copies. Similarly, many host-associated bacteria, such as *Mycobacterium leprae* and *Endomicrobium* spp. (Cole et al., 2000; Zheng et al., 2016) harbor large numbers of pseudogenes when compared to their free-living relatives (Lerat and Ochman, 2005). The surge in the numbers of IS elements and pseudogenes in recent pathogens and symbionts conforms with the expectations of the mutational hazard hypothesis: severe reductions in population size result in less effective selection, which promotes the accumulation of non-functional and slightly deleterious sequences. Note that the proliferation of IS elements and pseudogenes is observed only during the initial stages of genome reduction since these sequences will eventually be purged from the genome by mutational processes (Moran and Plague, 2004).

In contrast to IS elements and pseudogenes, the proportion of bacterial genomes occupied by prophages increases with genome size (Touchon et al., 2016), a surprising relationship given that population sizes are larger, and selection more effective, in bacteria with larger genomes. While prophages may occasionally encode beneficial functions, most of their genes are of no consequence to their bacterial host (Ptashne, 1992; Casjens, 2003) and are expected to be eliminated. However, bacteria harboring prophages could be favored in a competitive environment, since these elements can potentially be used to eliminate competitors (Brown et al., 2006). When considering all bacteria, the majority of genome size variation is due to the gain and loss of accessory genes (Touchon et al., 2009) whose functions are thought to help bacteria cope with different niches or lifestyle. That bacteria with larger population sizes accommodate more accessory genes could reflect the fact that large populations likely span more diverse ecological conditions and require larger gene repertoires (Juhas et al., 2009) or that larger populations experience more competition, since many accessory genes are now known to be involved in bacterial warfare (Wexler et al., 2016). Hence, accessory genes, and perhaps prophages, represent a diverse arsenal that allows bacteria to adapt to their ever-changing and competitive environments. The ability of a bacterial species to capture and maintain a diverse repertoire of accessory genes likely constitutes a key feature to occupying a wide range of environments and maintaining large population sizes.

Because bacteria can undergo frequent bouts of horizontal gene acquisition (HGT; Ochman et al., 2000), the genome contents and architecture of closely related strains within a bacterial species can vary in ways that are not apparent in eukaryotes. Members of the same eukaryote species typically do not vary in their gene repertoires, and the acquisition of functional sequences in eukaryotes rarely results from HGT (Keeling, 2009). These key differences between bacteria and eukaryotes help drive, in addition to their respective biases toward insertions and deletions, the evolution of genome sizes toward opposite directions when exposed to drift. Thus, bacterial genomes increase in size by aggregating adaptive gene modules when exposed to new selective pressures, whereas eukaryotic genomes increase in size by accumulating large amounts of non-functional DNA when exposed to drift.

## Author Contributions

Both authors, HO and L-MB, contributed equally to the conception, contents, and writing of this manuscript.

## Conflict of Interest Statement

The authors declare that the research was conducted in the absence of any commercial or financial relationships that could be construed as a potential conflict of interest.
